# Tobacco-Induced Oral Dysbiosis and Microbial Shifts: A Narrative Review of Their Role in Systemic Inflammation and Disease

**DOI:** 10.3390/microorganisms14051104

**Published:** 2026-05-13

**Authors:** Glenda M. Davison, Tandi Matsha, Shanel Raghubeer, Stanton Hector, Saarah Davids, Yvonne Prince

**Affiliations:** 1 SAMRC/CPUT Cardiometabolic Health Research Unit, Department of Biomedical Sciences, Cape Peninsula University of Technology, Symphony Way, Bellville 7535, South Africa; tandi.matsha-erasmus@smu.ac.za (T.M.); raghubeers@cput.ac.za (S.R.); hectors@cput.ac.za (S.H.); davidss@cput.ac.za (S.D.); 2 Vice Chancellors Office, Sefako Makgatho Health Sciences University, Ga-Rankuwa, Pretoria 0201, South Africa

**Keywords:** oral microbiota, periodontal disease, tobacco, smoking, systemic disease, inflammation, microorganisms, dysbiosis

## Abstract

The oral cavity is home to a diverse community of microbiota comprising bacteria, viruses, protozoa, and fungi. These microorganisms inhabit several oral niches and play a significant role in supporting both oral and systemic health. The fine balance between the microbial communities can be influenced by genetics and environmental factors, potentially leading to dysbiosis. Alterations in the oral microbiota have been implicated in periodontitis, chronic inflammation, and systemic disease. Tobacco has been identified as a major player in altering the oral microenvironment and disturbing the balance between potentially pathogenic and beneficial commensals. The resulting dysbiosis promotes inflammation and assists in the passage of pathogenic microorganisms into the blood system. This narrative review examines current evidence linking the use of tobacco with the dominance of pathogenic oral bacteria and a dysfunctional immune response. We explore how the chemicals and toxins in cigarettes promote a reduction in oxygen and cause changes in the abundance of anaerobic bacteria. After discussing the mechanistic pathways leading to periodontitis and the entry of microorganisms into the circulation, the review will interrogate previous studies and identify opportunities and priorities for future research.

## 1. Introduction

The human oral cavity is home to almost eight hundred bacterial species, fungi, viruses, and protozoa, all of which form a highly connected and complex ecosystem termed the oral microbiome [1,2,3]. The human body hosts several distinct microbiomes; the oral microbiome is the third largest after the gut and skin. Like other microbial communities, the oral biota interacts with the host immune system, forming a symbiotic relationship, and plays an essential role in maintaining human physiology and homeostasis [4]. In addition, these microorganisms cooperate closely with one another and create a finely balanced community, which contributes to promoting health and preventing infection.

Biofilms form an important part of this ecosystem and are formed when bacteria adhere to oral surfaces. Biofilms are complex, well-organized microbial communities that prevent colonization by pathogenic bacteria. They maintain homeostasis, contribute to immune regulation and assist in metabolic functions. Early colonizers, which are mainly beneficial commensals, initiate biofilm formation and rely on the chemical and physical forces of the oral surface to begin the process. Molecular interactions, such as Van Der Waals forces and Brownian motion, facilitate initial adhesion and an early microbial biofilm is formed. Other bacteria join this initial layer as the biofilm develops, thereby forming a complex and structured community. The microorganisms secrete several molecules, such as polysaccharides, proteins, and nucleic acids, collectively known as extracellular substances. These are important in protecting and maintaining the integrity of the microbial community. Biofilms are an essential part of a healthy oral cavity and are necessary to maintain the finely balanced ecosystem; however, pathogenic bacteria can become dominant and cause disease if not appropriately controlled [5].

Most individuals have a stable core microbiome that stays constant throughout life, as well as a variable biome. The variable biome is influenced by environmental factors, such as diet, lifestyle, genetics, medication, age, and physiology [6]. Any disturbances in the balance of the oral microbiota can lead to the emergence of pathogenic bacteria and the suppression of beneficial commensals. This dysbiosis has been identified as a critical factor in the development of gum disease; chronic inflammation; and systemic conditions, such as cardiovascular disease [7,8].

Tobacco smoke enters the body via the oral cavity and has been linked to seven million deaths globally, with most resulting from cardiovascular disease, lung cancer, and respiratory disorders [9]. Since the latter half of the 20th century, there has been a steady decline in the active smoking population, but recently, the World Health Organization (WHO) has estimated that 1.3 billion of the world’s population still smoke or use some form of tobacco, with 80% residing in low- or middle-income countries [10]. In South Africa, although tobacco smoking has also declined, in 2021, it was estimated that around 11.1 million, or 25.8% of the adult population, smoked [11]. Chronic long-term tobacco use predisposes individuals to the development of gingivitis and periodontitis, which have been linked to several systemic diseases [12,13]. Studies have proven that periodontal disease is not only isolated within the oral tissue but that pathogenic microorganisms can also enter the circulation and cause chronic inflammation, which leads to systemic conditions [14,15]. Among these, cardiovascular disease has been extensively studied and has been shown to have a strong relationship with periodontal disease [16].

This narrative review aimed to interrogate current literature investigating the impact of tobacco smoking on the oral microenvironment and the resulting shifts in microbiota. The review focused on the bacterial component of the biome and added studies from the genetically diverse populations of Southern Africa. It further probed findings on smoking-related immune dysregulation and the mechanistic pathways that contribute to the development of systemic disease.

## 2. Materials and Methods

A comprehensive literature search was performed for publications from 2005 to February 2026 using the PubMed, Google Scholar and ScienceDirect databases. The initial search identified 258 articles that included original research studies and review articles relevant to the topic. Keywords used to search the databases included “smoking”, “tobacco”, “cigarettes”, “oral biome”, “dysbiosis”, and “systemic disease”. A Boolean strategy, with words such as “And” and “Or”, was employed to refine and retrieve articles specific to the review.

Additional targeted searches were performed to identify original studies investigating tobacco-toxins and their effects on the oral environment published between 1995 and 2026, as well as recent population statistics (2020–2026) on the global and South African prevalence of smoking. These searches identified a further nine original research articles and five statements and fact sheets published by the World Health Organization and the South African Department of Health, resulting in a total of 272 manuscripts.

Non-peer-reviewed articles, duplicate manuscripts, preprints and articles focused on alternative forms of tobacco or E-cigarettes were excluded. The documents were further refined by excluding review articles containing substantial overlapping information. Following this process, 106 manuscripts remained, including 40 review articles, 62 original research articles, three fact sheets and 1 consensus report. The selected review articles comprised narrative reviews, systematic reviews and meta-analyses. The articles were subsequently grouped according to thematic subtopics, and information was extracted to formulate a comprehensive review of the literature. After extensive revision of the manuscript, a total of 104 manuscripts were included in the final review.

## 3. The Healthy Oral Microenvironment

The microorganisms that form part of the oral microbiome occupy several niches within the oral cavity. This includes both hard and soft tissue, such as the teeth, tongue, oral mucosa, palate, and tonsils [1,17,18,19,20]. A healthy oral microbiome is composed of aerobic and facultative anaerobic bacteria, which coexist symbiotically with other organisms and the host, thereby supporting health and preventing disease [21,22,23].

Research on the bacterial composition of the oral biome has demonstrated that the most predominant phyla are *Firmicutes*, *Proteobacteria*, *Bacteroidetes*, *Actinobacteria*, *Fusobacteria*, and *Spirochaetes* [17,24]. The genera and species derived from these phyla form well-balanced communities or biofilms on various oral surfaces and are supported by the surrounding microenvironment [5]. The most prominent genus is *Streptococcus* and *Actinomyces*, which are early colonizers in the establishment of biofilms [25,26]. Although considered beneficial, these microorganisms can become pathogenic in an abnormal environment, causing infections and gum disease [17,21].

The oral microenvironment, in a healthy state, is consistently controlled by several processes. The temperature is maintained between 35 and 36 °C, which is optimal for maintaining the delicate balance between microorganisms [1]. Saliva contributes to temperature control, assists with digestion, and provides moisture, which is essential for the biota to thrive. Importantly, saliva contains bicarbonate ions, phosphate, and proteins, which neutralize acids and support a stable pH of between 6.2 and 7.6 [27]. Furthermore, respiration facilitates a continuous flow of oxygen, a necessary requirement for aerobic bacteria to survive and to prevent colonization by pathogenic anaerobic bacteria. The expression of adhesion molecules and the secretion of biochemical substances allow microorganisms to exist in closely connected communities interacting with each other and the host immune system, preventing infection and maintaining homeostasis [22]. Beneficial organisms also contribute to digestion, detoxification, and maintenance of the mucosal barrier [24].

However, this stable ecosystem can change rapidly depending on many factors, including gene mutations, diet, age, drugs, and lifestyle habits, such as alcohol and smoking. The resulting alterations in microbial communities may lead to dysbiosis with potentially pathogenic bacteria becoming dominant and causing disease [17].

## 4. Tobacco-Induced Oral Dysbiosis

Tobacco and cigarette smoke contain a complex mixture of approximately seven thousand chemicals. Many are highly toxic, and around seventy are carcinogenic [28]. Several of these compounds exist in the tobacco plant, while others, including heavy metals, are present in soil and fertilizers. Other substances, such as nitrates, sugars, and proteins, are added during the processing of tobacco leaves [29]. Once the cigarette is lit and the tobacco is burned, toxic gases are released, including carbon monoxide, aromatic hydrocarbons, formaldehyde, and acrolein [30,31]. These toxins are known to cause DNA breakages, which have been directly connected to lung cancer and other malignancies [32]. The long-term accumulation of heavy metals leads to oxidative stress and a pro-inflammatory environment, which further contributes to systemic disease [33,34]. In addition, many of the toxins have antibiotic properties, which further promote microbial disruption and dysbiosis [28].

Nicotine and tar are the most dangerous compounds present in cigarettes. After being ingested or inhaled, nicotine binds to nicotinic acetylcholine receptors, which initiate the release of dopamine, creating feelings of pleasure and causing addiction [35]. Nicotine rapidly increases the heart rate and blood pressure, while reducing blood flow to the heart, which, over time, can result in heart disease [36]. Tar is formed after tobacco is burned and is a mixture of several chemicals, including carcinogens, which damage DNA. This compound coats the lungs; prevents the absorption of oxygen; and contributes to the development of Chronic Obstructive Pulmonary Disease (COPD), esophageal cancer, and other malignancies [32].

### 4.1. Disruption of the Oral Environment

The toxins and chemicals from tobacco smoke enter the body via the oral cavity. They change the oral microenvironment and promote the proliferation of pathogenic bacteria [37]. After combustion of tobacco, the temperature within the oral cavity quickly rises, and the flow of saliva is reduced, which compromises pH control and promotes an acidic environment [38,39]. Furthermore, the toxins initiate a shift in the residing microbiota, which leads to the release of acidic metabolically derived compounds and a resultant drop in pH [38,39]. The tobacco smoke further induces vasoconstriction within the microvessels of the gingival tissue. The release of carbon monoxide and other substances reduces oxygen tension, creating an anaerobic environment and the formation of reactive oxygen species (ROS) [33]. Together, these alterations favor the expansion of anaerobic pathogenic microorganisms, while beneficial bacteria, which are important in maintaining health and oral homeostasis, are suppressed [31].

**Figure 1:**
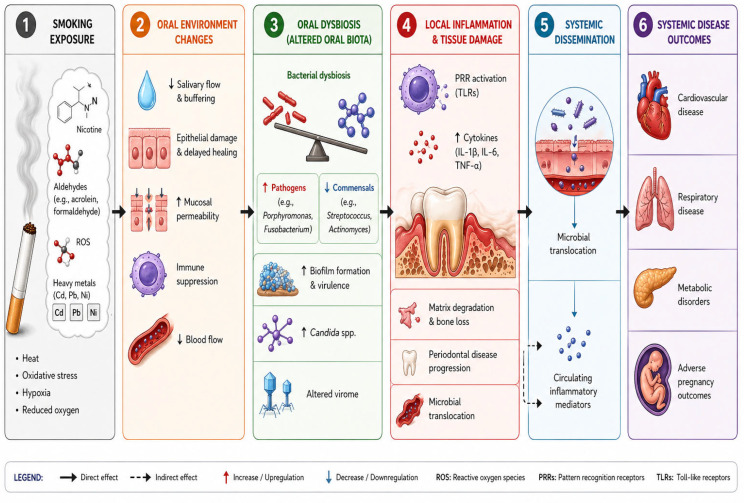
This flow diagram demonstrates how cigarette smoke alters the oral microenvironment to cause dysbiosis, local immune dysfunction, gum disease, and systemic inflammation. In panel one, the toxic chemicals and gases released from the burning tobacco cause reduced oxygen flow, heat, and oxidative stress. The changes in the oral microenvironment caused by the smoke are shown in panel two. These changes include reduced salivary and blood flow, epithelial damage, and immune suppression, which result in poor healing. In panel three, the effects of these environmental changes result in a disruption in the balance of bacteria and other microorganisms, such as fungi and viruses. This dysbiosis leads to a higher relative abundance of pathogenic organisms, suppression of beneficial commensals, and virulent biofilm formation. Subsequent inflammation and gum disease are depicted in panel 4. Bacterial toxins activate the innate immune response, resulting in the release of proinflammatory molecules, tissue damage, gingivitis, and periodontitis. Panel 5 illustrates how periodontal disease causes epithelial damage, facilitating the movement of bacteria into the circulation, causing low-grade inflammation and increasing the risk of systemic disease. Panel 6 demonstrates the outcome of this process and how the cycle of chronic inflammation caused by circulating microorganisms can affect distant organs.

Parallel to these changes, ROS, nicotine, and other toxic substances alter the composition of saliva, damage the epithelial barrier, and modify the immune response [40,41,42]. The combination of these changes establishes a pro-inflammatory microenvironment, which has a reduced capacity to clear infection and contributes to an increased risk of periodontal disease (Figure 1).

### 4.2. Microbial Dysbiosis and Shifts in Diversity Caused by Tobacco

The bacterial shifts caused by tobacco smoking across the phylum, genus, and species levels have been well documented [43,44,45,46,47,48,49,50,51,52]. These changes affect the well-balanced microbial communities, creating conditions for pathogenic bacteria to become dominant. Studies have been heterogeneous and often conflicting due to several influencing factors. However, most researchers consistently report a higher relative abundance of pathogenic anaerobic bacteria and a reduction in beneficial commensals.

At the phylum level, the most consistent findings in tobacco users are a higher relative abundance of *Firmicutes* and *Actinobacteria* with a reduction in *Proteobacteria* [43,44,45,46,47,48,49,50,51]. In contrast, a recent study from South Africa reported a lower abundance of *Actinobacteria* in smokers [52] (Table 1). This study was performed on a Southern African mixed ancestry community of low to middle income using subgingival plaque. The ethnicity and sample type differed from those of other studies and may explain differences in the observed results. Interestingly, the role of genetic diversity has been debated. In another study from Southern Africa, the composition of the oral microbiota from several genetically diverse populations was investigated. The authors reported significant variations in the microbial profiles but concluded that this was not related to ethnicity or livelihood but rather socioeconomic factors and poor health conditions [53]. In contrast to this, a study including low-income African Americans and Europeans reported significant differences between the two population groups. However, the effects of smoking were similar [48]. These articles emphasize the influence of the environment, health, and population characteristics on studies of the oral biome and emphasize the need for more standardized and representative research.

At the genus and species levels, results are more inconsistent and are probably influenced by differing study designs, methodologies, sample types, and collection sites. Although most recent studies use 16S rRNA sequencing for analysis, there are differences in which hypervariable region of the gene is sequenced, the sequencing platform used, and the bioinformatics analysis [37,51]. These differences can all influence the results and final interpretation. In addition to methodology, sample sizes and the type of tobacco also influence the outcome [37]. The articles analyzed in this review describe varying sample sizes, ranging from fifty [46] to over four thousand [33]. In clinical research, small sample sizes can reduce the statistical power of a study, potentially leading to biased or non-representative results.

In this review, most studies consistently reported differences between smokers and non-smokers. Common to most reports was an increased relative abundance of *Prevotella*, *Veillonella*, *Streptococcus*, *Actinomyces*, and *Fusobacterium* with a depletion of *Neisseria*, *Haemophilus*, and *Lautropia* [33,45,47,54]. Although inconsistencies were observed at the species level, there is general agreement that smoking results in the growth of anaerobic bacteria, such as *P. gingivalis*, *F. nucleatum*, and *T. denticola*, which are important in biofilm formation [31,52,55]. The differences between “current” and “never” smokers were also investigated. These findings suggest that former smokers and individuals who have never smoked display a similar bacterial profile, indicating that the oral biota have the capacity to recover [48,50,51].

Although the evidence for tobacco-associated oral biota changes is moderately strong at the phylum and genus levels, species-level shifts and diversity measurements are less consistent across studies (Table 2).

#### Alpha and Beta Diversity

Alongside the shifts in bacterial communities, it is standard to document microbial diversity. Alpha diversity measures the variation in bacteria in a single sample and indicates the richness and evenness of the microbes present [26].

Studies measuring alpha diversity in tobacco users are often inconsistent and are influenced by several factors [56]. One explanation is that bacterial diversity and composition vary significantly between oral niches, such as saliva, the tongue, subgingival plaque, the palate, and the tonsils. In support of this, a recent study compared the bacterial composition in swabs taken from the buccal mucosa, the tongue, the palate, and the floor of the mouth. 16S rRNA sequencing was used, and the results showed that the samples from the palate and tongue had higher alpha diversity scores compared to the other two sites. The bacterial communities also differed depending on the sample site. For example, buccal mucosa and the floor of the mouth had a higher relative abundance of *Streptococcus*, while the tongue exhibited greater levels of *Neisseria* and *Rothia* [57]. One advantage of this investigation was the standardized methodology used for all samples, thus enabling reliable comparisons. In support, further studies contrasting samples from the saliva and tongue reported differences in both the alpha diversity and microbial profiles between the two sample types [58]. Both examples emphasize the complexity of studying oral microbial shifts and the importance of being aware of the sampling site and type.

In a systematic review of thirty-six articles examining the effect of tobacco on the oral biota, most articles reported an increased alpha diversity in smokers. However, five observed no difference, while four observed a reduction. Despite these differences, all studies demonstrated significant changes in the microbiota in those who smoked [37]. This systematic review highlighted the heterogeneity of study designs, sample types, and methodology as limitations and emphasized the need for more standardized research.

A further explanation for differing measurements is that microbial diversity decreases with advancing age. This was clearly demonstrated in a large study of 4387 adults between 30 and 69 years of age. Both alpha and beta diversity decreased significantly with age, particularly in smokers over 60 years [33].

Unlike alpha diversity, beta diversity determines the differences between groups (e.g., smokers vs. non-smokers). Beta-diversity measurements are more consistent than alpha diversity [59].

In this review, we grouped studies according to sample type and examined the variation in both alpha and beta diversity. Although all studies reported a significant difference in beta diversity, the alpha diversity varied significantly and was not dependent on sample type (Table 1). This variation in alpha diversity could be explained by factors, such as sample size and population group, which differed significantly across all studies. This analysis emphasized the importance of reporting both alpha and beta diversity alongside the microbial profile to improve interpretation and arrive at realistic conclusions.

In summary, while most studies report microbial alterations towards anaerobic pathogenic bacteria in smokers, alpha diversity measurements are inconsistent and should be interpreted with caution as they are influenced by several factors, including sampling sites, population diversity and methodology (Table 2).

### 4.3. Tobacco Alters Immune Function

Parallel to the altered microbiota, tobacco can also disrupt immune function. The toxins within tobacco smoke impact both innate and adaptive immunity [40]. The release of toxic chemicals, including nicotine, reactive nitrogen species (RNS), ROS, and free radicals, causes systemic oxidative stress and leads to chronic inflammation. Mechanistic studies using animal models have demonstrated that this is achieved through the stimulation of MAPK signaling pathways, which go on to activate transcription factor pathways, such as nuclear factor kappa B (NFkB). NFkB proteins play a significant role in initiating inflammation. They move into the nucleus of the cell and trigger the transcription of pro-inflammatory genes [42]. In addition to chronic inflammation, nicotine can have anti-inflammatory effects by binding to nicotinic acetylcholine receptors (nAChRs) [60]. This results in a dysfunctional immune response, which results in poor mouth infection clearance and wound healing. Together, these increase the risk of gingivitis and periodontitis.

## 5. Microbial Dysbiosis and Inflammation in Periodontitis

Oral microbial dysbiosis favors anaerobic bacteria, such as *P. gingivalis* and *F. nucleatum*, which form part of multi-species biofilms situated in the gingival sulcus between the tooth and the gum [61]. Gram-positive bacteria are often the early colonizers and create an environment that attracts secondary and late colonizers, which are often Gram-negative. As the biofilm continues to develop, nutrients are still able to enter, and the bacteria are protected from mechanical removal and the host immune response [5,24,62].

Several studies have shown that as the oral environment becomes more anaerobic, bacterial components, including lipopolysaccharide, butyrate, leukotoxin, hydrogen sulfide, and gingipains, are released into the gingival tissue and stimulate the innate immune response. Innate mediators, such as complement, neutrophils, macrophages, and natural killer cells, are activated. Bacterial toxins bind to Toll-like receptors 2 and 4, expressed on monocytes and granulocytes, leading to the secretion of pro-inflammatory cytokines. These include tumor necrosis factor α (TNF-α); C-reactive protein; interleukins-1, -6, -8, and -17; and nuclear factor kappa B ligand (RANK-L) [63,64,65]. The persistent release of these molecules results in a vicious cycle of immune activation, which eventually causes immune exhaustion and dysregulation.

This situation is compounded by nicotine, which has been shown to impair neutrophil function, skew monocyte phenotype towards the proinflammatory M1 subtype, and cause bone loss [65,66,67]. Nicotine increases neutrophil chemotaxis but impairs phagocytosis and the killing of pathogens. Evidence suggests that nicotine can cause the release of granule content and stimulate neutrophil extracellular traps (NETS). However, this does not improve bacterial killing but rather exacerbates inflammation and tissue damage [68]. The anaerobic environment prevents neutrophils from producing ROS and superoxides, which further inhibit the host from clearing infections and increase the risk of periodontitis [69].

Pro-inflammatory cytokines, matrix metalloproteinases (MMPs), and osteoclast activating factors are continually released and eventually result in the breakdown of connective tissue with the development of periodontal pockets. This favors further inflammation and tissue destruction by creating a gateway for bacteria and inflammatory mediators to enter the circulation [70].

## 6. Microorganisms Enter the Circulation and Cause Systemic Disease

Periodontitis, inflammation, and routine activities, such as eating and brushing teeth, can disrupt the epithelial barrier. In addition, bacterial components break down junctional adhesion molecules between the epithelial cells. This facilitates the movement of bacteria into the capillaries surrounding the tooth [41], leading to low-grade systemic inflammation. Circulating bacteria, pro-inflammatory cytokines, and cells can influence distant organs and increase the risk of developing systemic disease (Figure 1). Several reports have linked periodontitis to diseases such as diabetes [71], cardiovascular disease [16], malignancy, autoimmune disorders [72], and Alzheimer’s disease [73].

### 6.1. Cardiovascular Disease

The association between periodontal disease and cardiovascular disease is well documented [16]. Once bacteria enter the peripheral blood, they can avoid removal by the immune system and continue to fuel an inflammatory environment and oxidative stress. Cardiovascular disease can be caused by bacteria either directly invading arterial cell walls or by driving persistent inflammation and causing endothelial damage [7,12].

Clinical evidence supporting a causal relationship is inadequate, and the mechanisms leading to cardiovascular disease and myocardial infarction require further investigation. Researchers have attempted to study microbial profiles in the peripheral blood of individuals with cardiovascular disease. These have revealed an increase in diverse circulating microorganisms compared to controls [74,75,76]. However, these studies have been cross-sectional, with small sample sizes, and have been unable to establish the source of these organisms. These could have originated from the gut, oral cavity, or skin biome.

Mechanistic studies using well-characterized animal models have explored the effects of specific oral bacterial antigens from *P. ginivalis* and *F. nucleatum* on endothelial dysfunction and immune regulation. These experiments have shown that bacterial components do cause endothelial dysfunction or systemic inflammation. Experiments have shown that the bacterial components activate pro-inflammatory B2-cells within lymph nodes and can induce a Th1 inflammatory response [77,78]. This evidence sheds light on the mechanisms leading to inflammation and cardiovascular disease; however, additional comprehensive human studies are required.

Pathogenic bacteria (e.g., *P. gingivalis*) and their components can enter endothelial cells lining vessel walls, thereby promoting injury and eventual apoptosis [79]. Persistent inflammation and oxidative stress cause damage to the endothelium and promote the expression of adhesion molecules, such as intercellular adhesion molecule 1 (ICAM-1) and vascular cell adhesion molecule-1 (VCAM-1). Activated circulating monocytes and neutrophils bind to these receptors and attract others to the inflamed vessel wall [80]. Monocytes, attracted by the secretion of chemokines, migrate across the endothelial cell layer, differentiate into macrophages, and phagocytose oxidized low-density lipid proteins (ox-LDL). Once this has occurred, the macrophages transform into foam cells, which is the first step in the development of an atherosclerotic plaque and cardiovascular disease [79,81].

Together, these studies support the role of pathogenic bacteria moving from the oral cavity to the peripheral blood and causing inflammation, endothelial dysfunction and atherosclerosis. However, the specific role of tobacco-associated dysbiosis remains less defined, as the current evidence is mostly associative and does not demonstrate a causal relationship.

### 6.2. Gastrointestinal Disorders and the Gut–Oral Axis

The oral cavity is the entrance to the digestive system, and both normal and potentially pathogenic oral bacteria can move into the gut and cause gut dysbiosis [8,82]. Shifts in the gut microbial ecosystem lead to inflammation and can increase the risk of diseases, such as inflammatory bowel disease and irritable bowel syndrome [82].

*P. gingivalis* and *F. nucleatum*, both common bacterial species found in the oral cavity of smokers, have been associated with oral carcinoma and colorectal cancer [83,84]. In the colon, both species create a pro-inflammatory environment and dysregulate the immune response, contributing to malignant transformation.

In vitro and animal studies have clarified how *F. nucleatum* can initiate colorectal cancer. The adhesion molecules FadA and Fap2 are expressed on the bacterial surface. FadA attaches to the receptor E-cadherin on the epithelial cells of the intestine. This interaction activates β-catenin signaling pathways, which promote the proliferation of tumor cells. Meanwhile, Fap2 inhibits the activation of natural killer cells and CD8+ cytotoxic T cells, allowing tumor cells to evade immunity and proliferate [85,86]. Studies have also demonstrated that bacteria, including *F. nucleatum,* can directly activate oncogenes, such as *Myc* and *cyclin D1*, initiating the process of oncogenesis and driving uncontrolled proliferation of transformed cells [86]. These findings imply that the process of oncogenesis is complex. A series of events causes the proliferation of malignant cells, with pathogenic bacteria from the oral cavity contributing to this process.

### 6.3. Diabetes Mellitus

Metabolic disorders, such as diabetes, have also been associated with periodontitis. The relationship between diabetes and periodontitis has often been described as bi-directional [87]. It has been reported that individuals who have periodontitis have a 26% increased risk of developing diabetes, while those with diabetes have a 24% chance of developing periodontitis [88]. Both conditions are complex and cause chronic inflammation, and it is accepted that diabetes and periodontitis interact with each other, leading to worsening of both diseases [15,87]. In support, a recent meta-analysis of eleven studies examined the effects of periodontal treatment on the severity of diabetes. This study was able to show that after successful removal of the periodontal infection and lowering of oral inflammation, a significant reduction in HbA1c and C-reactive protein levels was observed at 3 and 6 months post-treatment [89].

The relationship between these two diseases is multifaceted, and it is agreed that the link is related to a combination of elevated glucose levels in diabetes and sustained inflammation, which is common to both conditions [71]. Uncontrolled levels of glucose accelerate the formation of advanced glycation end products (AGEs) and inhibit the function of neutrophils and the immune response. Neutrophils are important in clearing infections and wound healing, and therefore, their dysfunction contributes to the worsening of gum disease [90,91,92]. In contrast, periodontitis leads to the secretion of cytokines such as Il-1, Il-6, and TNF-a, which intensifies inflammation and worsens insulin resistance [93]. This negative feedback loop leads to a deterioration in both conditions.

Tobacco use exacerbates the situation by altering the microbial environment and causing further immune dysfunction. When periodontitis, diabetes, and smoking are all present, the vicious cycle between the two diseases is accelerated.

### 6.4. Other Systemic Diseases

The disruption of the oral microbiota by tobacco smoking has been associated with several other systemic diseases. However, most research is observational, and further comprehensive research is required. One example is rheumatoid arthritis (RA), which is an autoimmune disorder characterized by chronic inflammation. Recent research examining the serum of patients with RA has revealed elevated antibody levels specific to pathogenic bacteria originating from the oral cavity (e.g., *P. gingivalis*) [94]. This has led to the proposal that the entry of bacteria into the circulation may initiate inflammation and a possible autoimmune response. Neurodegenerative disorders, such as Alzheimer’s and Parkinson’s disease, have also been associated with inflammation and oral dysbiosis. Although research is still in the initial stages, it has been established that systemic inflammation in those with periodontitis leads to accelerated progression and worsening of both conditions [95].

A further example of how gum disease can cause systemic conditions is the evidence linking periodontitis with adverse pregnancy outcomes. Pregnancy and smoking both alter the oral microbiome [96]. Women who develop periodontitis during pregnancy have an increased risk of pre-eclampsia, pre-term births, and underweight infants [97]. However, recent research has shown that the effects of pregnancy and smoking on the oral microbial profiles are different and distinct. Unlike those who smoke, pregnant women have lower levels of Gram-negative bacteria [96]. However, the changes and resulting dysbiosis occurring in pregnant women result in inflammation and a dysfunctional immune response, which is amplified when both pregnancy and smoking are present together [97].

The association between smoking-induced oral dysbiosis and systemic disease is often reported; however, most evidence is derived from either cross-sectional or animal studies, and a causal relationship remains unclear.

## 7. Oral Microbiome Recovery Following Smoking Cessation

The toxins and gases released by tobacco smoking have significant effects on the oral environment and the microbial communities residing in the oral cavity [98]. Despite the potential damage to periodontal tissue, researchers comparing the oral microbiota of former smokers with individuals who have never smoked have demonstrated that healthy microorganisms have the potential to recover after tobacco use is discontinued [48,50,51]. Improvements in the flow of saliva, oxygenation, and pH stabilization can be observed within 2–6 weeks. Once the environment is restored, normal aerobic bacteria can reoccupy the oral niches and restore balance in the microbial communities. In a small longitudinal study, eleven individuals who stopped smoking were followed up over 12 months to observe changes in oral bacterial communities. Despite the small numbers, researchers observed that periodontal therapy induced a significant reduction in pathogenic bacteria and inflammation and shifted the microbiota toward a healthy state [99].

## 8. Limitations and Future Research

Although there is increasing evidence indicating a risk of systemic diseases linked to smoking-related oral dysbiosis, most clinical studies demonstrate an association rather than a causal relationship. Many of the published studies are cross-sectional and observational, with a lack of longitudinal data, which could track the progression of the disease over time. To address these challenges, multi-omics approaches are required to fully clarify the mechanisms leading to systemic conditions. This may include detecting shifts in microbial communities linked to specific conditions and the identification of possible oral biomarkers, which could predict the risk of disease development. Alternatively, these microbial signatures could be used to formulate targeted therapies to prevent or treat associated conditions [100,101,102]. As the toxins and chemicals in cigarettes can cause systemic inflammation and disease, further research on the direct effects of these compounds on the immune system and organs is required.

A major limitation of previous research is the insufficient control of confounding factors, such as lifestyle, dental hygiene, diet, age, and alcohol intake. These all cause dysbiosis and disease, making interpretation and conclusions difficult. Compounding this is the lack of standardization in methodology, study designs, sample type, and sample size [37]. A further inconsistency and barrier to reliable interpretation is the variation in geographical regions and population groups. Ethnicity, socioeconomic factors and access to health care can influence the development of periodontitis and systemic disease [53]. To obtain a more consistent picture of tobacco-induced alterations to the oral biome, large, collaborative, standardized investigations, utilizing the same study design and methodology, are recommended. Confounding factors, such as age, sex, type of tobacco, diet, and lifestyle, would need to be recorded and accounted for during statistical analysis to obtain realistic and comparable results.

One limitation of this narrative review is that it concentrated on the bacterial components of the oral biome. However, there is growing evidence that viruses, parasites, and fungi can also initiate inflammation. Although there is less evidence, in vitro and animal studies have established that smokers are at higher risk of developing fungal infections, such as oral candidiasis, which can lead to periodontitis and systemic inflammation [38,103]. Further research adopting comprehensive approaches, including cell culture, microscopy and multi-omics techniques, is needed to understand the complex interactions of these microorganisms and how they contribute to the pathogenesis of disease.

As traditional cigarette smoking declines, the popularity of E-cigarettes and vaping has increased, especially among the youth. This review did not address these products; however, there is growing evidence that the aerosols produced from E-cigarettes contain several toxic compounds [67]. These chemicals disrupt the oral microbiome and promote the growth of opportunistic pro-inflammatory organisms. Vaping has been shown to disrupt the epithelial barrier, impair the immune response, and cause oxidative stress. These changes can lead to chronic systemic inflammation [104]. Like the analysis of traditional cigarette smoking, standardized studies are required to account for the variation in products, including chemical composition, flavorings, and nicotine concentration.

Finally, there is a lack of longitudinal studies that investigate changes in the oral biota over time, particularly after the cessation of smoking. These studies should include the time to full recovery and the impact of improved oral hygiene, probiotics, and prebiotics on the restoration of healthy micro-organisms [100].

Despite the limitations, several findings in smokers compared to non-smokers are consistent and robust. These include the shift to Gram-negative anaerobic bacteria and the relative increased abundance of genera including *Prevotella*, *Veillonella* and *Fusobacterium.* The significant differences in beta diversity are similar across all studies; however, alpha diversity measurements and changes at the species level are diverse and inconsistent, making interpretation difficult. These results are most likely influenced by study designs, sampling sites, population differences and methodology approaches, highlighting the need for large standardized collaborative studies.

## 9. Conclusions

This review provides a mechanistic integrated synthesis of literature linking tobacco-related changes to the oral microenvironment and dysbiosis, immune dysregulation and systemic disease. Unlike previous reviews, which focus largely on microbial shifts and periodontal inflammation, this review critically examines how the environment, the host and microorganisms converge to initiate systemic disease. It further advances the topic by providing data from diverse, underrepresented Southern African populations, demonstrating the importance of socioeconomic and environmental factors in the variability of oral microbial profiles.

Microbial changes in smokers are well documented, but the mechanistic pathways linking these alterations to systemic disease remain poorly understood. Addressing this gap will require well-coordinated interdisciplinary research. Advances in this area are essential for the identification of early microbial biomarkers and the development of targeted therapeutic approaches.

## Figures and Tables

**Table 1:** Summary of studies investigating the impact of cigarette smoking on the oral microbiota.

Ref	Study (Author, Year)	Number	Sample Type	Diversity (α/β)	Main Phyla Reported	Main Findings in Smokers
[43]	Huang et al., 2023	316	Saliva	↑/ Significant	Firmicutes ↑ Bacteroidetes ↑ Proteobacteria ↓	Altered microbiota & inflammation-associated taxa
[44]	Chattopadhyay et al., 2024	150	Saliva	↔/ Significant	Firmicutes ↑ Bacteroidetes ↑	Dysbiosis; increased anaerobes & reduced commensals
[45]	Al-Zyoud et al., 2019	105	Saliva	↓/ Significant	Firmicutes ↑ Actinobacteria ↑ Proteobacteria ↓	Distinct microbial profiles with increased pathogenic taxa
[46]	Rodríguez-Rabassa et al., 2018	50	Saliva	NR	Bacteroidetes ↑ Firmicutes ↑	Altered microbial composition; increased pro-inflammatory cytokines
[48]	Yang et al., 2019	1616	Oral rinse	Mixed/ Significant	Firmicutes ↑ Actinobacteria ↑ Proteobacteria ↓	Altered microbiota across populations; consistent enrichment of pathogenic taxa
[49]	Beghini et al., 2019	282	Oral rinse	↔/ Significant	Firmicutes ↑ Bacteroidetes ↑	Shift toward bacteria with altered oxygen utilization; increased anaerobic profile
[51]	Wu et al., 2016	1204	Oral wash	↑/ Significant	Firmicutes ↑ Bacteroidetes ↑ Proteobacteria ↓	Significant microbial differences; enrichment of pathogenic anaerobes
[50]	Sato et al., 2020	92	Tongue	↑/ Significant	Bacteroidetes ↑ Fusobacteria ↑	Enrichment of anaerobic species
[52]	Prince et al., 2024	128	Subgingival plaque	↔/ Significant	Firmicutes ↑ Bacteroidetes ↑ Actinobacteria ↓	Altered subgingival microbiota in a South African cohort

Abbreviations: α, alpha diversity; β, beta diversity; ↑, increased; ↓, decreased; ↔, no significant change; NR, not reported.

**Table 2:** Summary of consistency and strength of evidence in studies of tobacco-associated oral dysbiosis.

Finding	Consistence	Interpretation & Influencing Factors
Abundance of anaerobic bacteria	High	Robust finding
Phylum changes	Moderate	Population differences
Genera-level shifts	Moderate	Sequencing method, sample site, study design.
Species-level shifts	Low	Methodology, sample site, sample type, study design
Beta diversity differences	High	Reliable findings
Alpha diversity measurements	Low	Sample site, method, population diversity, age

## Data Availability

No new data were created or analyzed in this study. Data sharing is not applicable to this article.
